# New Remedies—McKesson & Robbins

**Published:** 1887-09

**Authors:** 


### *NEW REMEDIES—McKESSON & ROBBINS.*

We would call the attention of our readers to the new remedies and elegant preparations offered to the profession by McKesson & Robbins. As will be seen in the closing lines of their advertisement in this issue, thev offer to send to any physician asking for them, monographs giving the most recent observations as to the use of various new remedies. We would especially advise a trial of the new drug Salol, which they offer in convenient form for administration, and their label is a guarantee of purity. Salol, or Salicylate of Phenol, is composed of 40 per cent. of carbolic acid and 60 per cent. of salicylic acid. While rather more efficient than salicylic acid or salicylate of soda, it possesses the great advantage of being tasteless and in not producing any gastric disturbance.

In the October (1886) number of the JOURNAL, we gave a translation of the earliest reports on the use of the new drug, and since then experience has fully confirmed these observations.

---

WE call the attention of physicians and druggists to the new law, relative to the refilling of a certain class of prescriptions.

We believe that many patients have become victims of the opium habit from the facility with which they could have prescriptions containing anodynes refilled. The remedy for this evil will be had in the enforcement of the new law:

AN ACT to regulate the sale of morphine by druggists and apothecaries in this State. Passed June 21, 1887; three-fifths being present.

*The People of the State of New York, represented in Senate and Assembly, do enact as follows:*

SECTION 1. From and after the passage of this act, no pharmacist, druggist, apothecary or other persons shall re-fill, more than once, prescriptions containing opium or morphine or preparations of either in which the dose of opium shall exceed one-fourth grain or morphine one-twentieth grain except with the verbal or written order of a physician.

SEC. 2. Any person violating the provisions of section one of this act shall be deemed guilty of a misdemeanor, and shall, upon conviction thereof, be fined not less than ten dollars nor more than twenty-five dollars, in the discretion of the court, for each and every such offense.

